# OmicShare tools: A zero‐code interactive online platform for biological data analysis and visualization

**DOI:** 10.1002/imt2.228

**Published:** 2024-08-01

**Authors:** Hongyan Mu, Jianzhou Chen, Wenjie Huang, Gui Huang, Meiying Deng, Shimiao Hong, Peng Ai, Chuan Gao, Huangkai Zhou

**Affiliations:** ^1^ Product Research and Development Center Guangzhou Genedenovo Technology Co. Ltd. Guangzhou China

## Abstract

The OmicShare tools platform is a user‐friendly online resource for data analysis and visualization, encompassing 161 bioinformatic tools. Users can easily track the progress of projects in real‐time through an overview interface. The platform has a powerful interactive graphics engine that allows for the custom‐tailored modification of charts generated from analyses. The visually appealing charts produced by OmicShare improve data interpretability and meet the requirements for publication. It has been acknowledged in over 4000 publications and is available in https://www.omicshare.com/tools/.

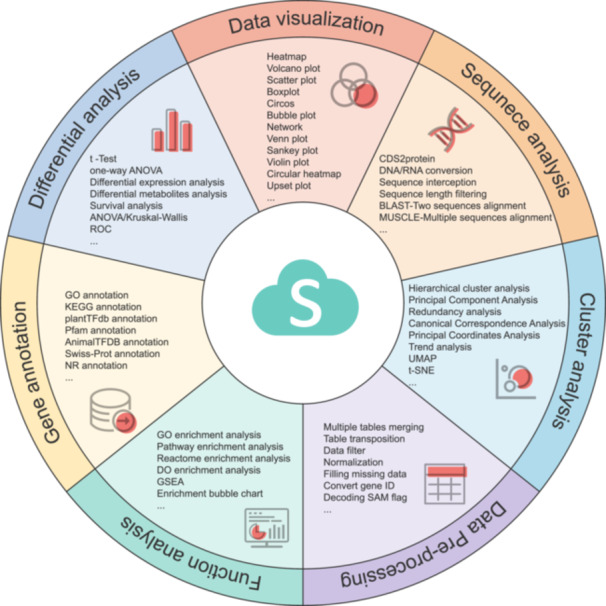

Advances in high‐throughput sequencing technologies have resulted in an exponential increase in the volume of biological data, creating significant challenges for biologists in analyzing extensive data sets and extracting meaningful biological insights from them [1]. Typically, the process from initial data acquisition to the dissemination of research findings involves three key stages: statistical data analysis, the creation of statistical charts, and graphical enhancement. Many bioinformatics platforms or tools for high throughput omics data, such as Majorbio Cloud [2], SangerBox [3], ImageGP [4], Wekemo [5], Evenn [6], Circos in TBtools [7], complexHeatmap [8], and ggVennDiagram [9], have emerged. Most of these platforms were developed for specific omics research, especially metagenomics and transcriptomics, and some tools provide solutions for special problems. However, the default graphic outputs from these platforms or tools often require extensive fine‐tuning using graphic editing software (such as Adobe Illustrator or Photoshop) to attain a standard suitable for publication.

Furthermore, many tools for analyzing and visualizing biological data lack user‐friendly interfaces, which makes the process more complicated rather than simple mouse clicks [10]. To bridge this gap and empower all biological researchers, including those without programming skills, to smoothly navigate through data analysis, visualization, and graphic refinement in one continuous workflow, we present the OmicShare tools platform (https://www.omicshare.com/tools/). This platform acts as a zero‐code, interactive portal that streamlines data analysis, granting researchers the capability for easy, adaptable, and individualized exploration of their data.

## OVERVIEW OF OMICSHARE TOOLS

**Figure 1:**
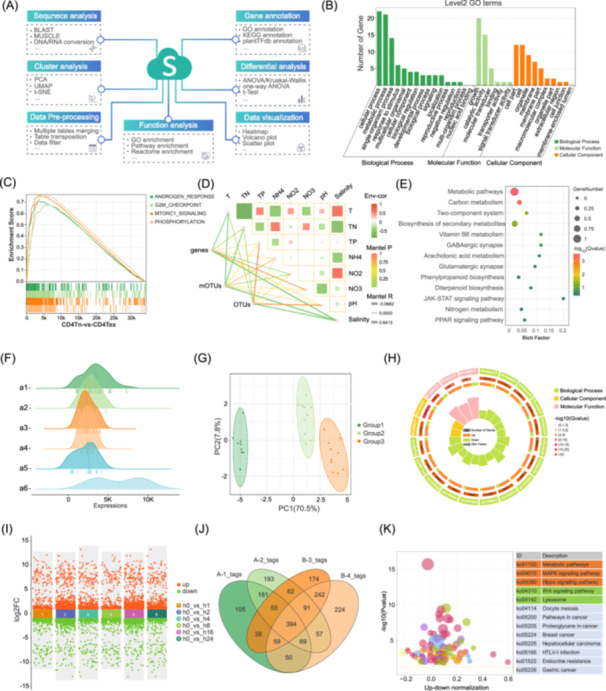
Outline of functions in OmicShare tools and examples of output. (A) The classification of OmicShare tools; (B) Gene Ontology (GO) enrichment bar plot; (C) Gene Set Enrichment Analysis Enrichment; (D) network heatmap; (E) bubble plot; (F) ridgeline plot; (G) principal component analysis (PCA) scatter plot; (H) enrichment circle; (I) scatter plot; (J) Venn plot; (K) enrichment bubble plot. ANOVA, analysis of variance; BLAST, basic local alignment search tool; FC, fold change; KEGG, Kyoto Encyclopedia of Genes and Genomes; MUSCLE, multiple sequence comparison by log‐expectation; t‐SNE, t‐distributed stochastic neighbor embedding; UMAP, uniform manifold approximation and projection.

The OmicShare tools suite is a robust collection encompassing 161 bioinformatic utilities designed to address the multifaceted needs of biological research. These utilities span a wide array of functions, from nucleic acid and protein sequence analysis to gene function annotation, differential expression assessment, clustering evaluation, and custom graphical output creation. This rich assortment offers a full suite of methods for the meticulous analysis and visualization of omics data, as depicted in Figure 1A. Within this framework, researchers can effortlessly carry out gene function enrichment analysis utilizing databases like the Gene Ontology (GO), Kyoto Encyclopedia of Genes and Genomes (KEGG), Disease Ontology, and Reactome. In addition, the platform encompasses a comprehensive set of statistical tests, such as correlation, variance, and principal component analysis (PCA), together with survival analysis. It also facilitates the production of publication‐ready graphics, including heatmaps, Venn diagrams, and scatter plots, that cater to the high standards of scientific communication.

Within this comprehensive suite, 129 tools offer data visualization features that simplify the creation of standard scientific imagery and enable researchers to conceive custom visualizations. Examples of such tools include those for generating various specialized diagrams: grouped bar charts for GO enrichment, correlation network heatmaps, composite plots for Gene Set Enrichment Analysis, PCA scatter plots with confidence intervals, scatter diagrams for comparing multiple cohorts, and ridge plots, as displayed in Figure 1B–K.

Additionally, the graphical outputs from the integrated OmicShare tools suite are exceptionally diverse and detailed. For instance, the KEGG enrichment analysis tool does not just generate standard bar charts and bubble charts (Figure 1E), it also creates more specialized visuals like circular diagrams and bubble diagrams (Figure 1H,K), offering a variety of ways to display data. To aid in data management and adhere to the formatting needs of various analyses, the OmicShare tools include a suite of data format conversion utilities. These tools streamline the process of data normalization and standardization, facilitating the conversion of gene IDs across different nomenclatures in various species and accommodate the transformation between “long” and “wide” data formats for ease of analysis.

### User‐friendly tool interface

**Figure 2:**
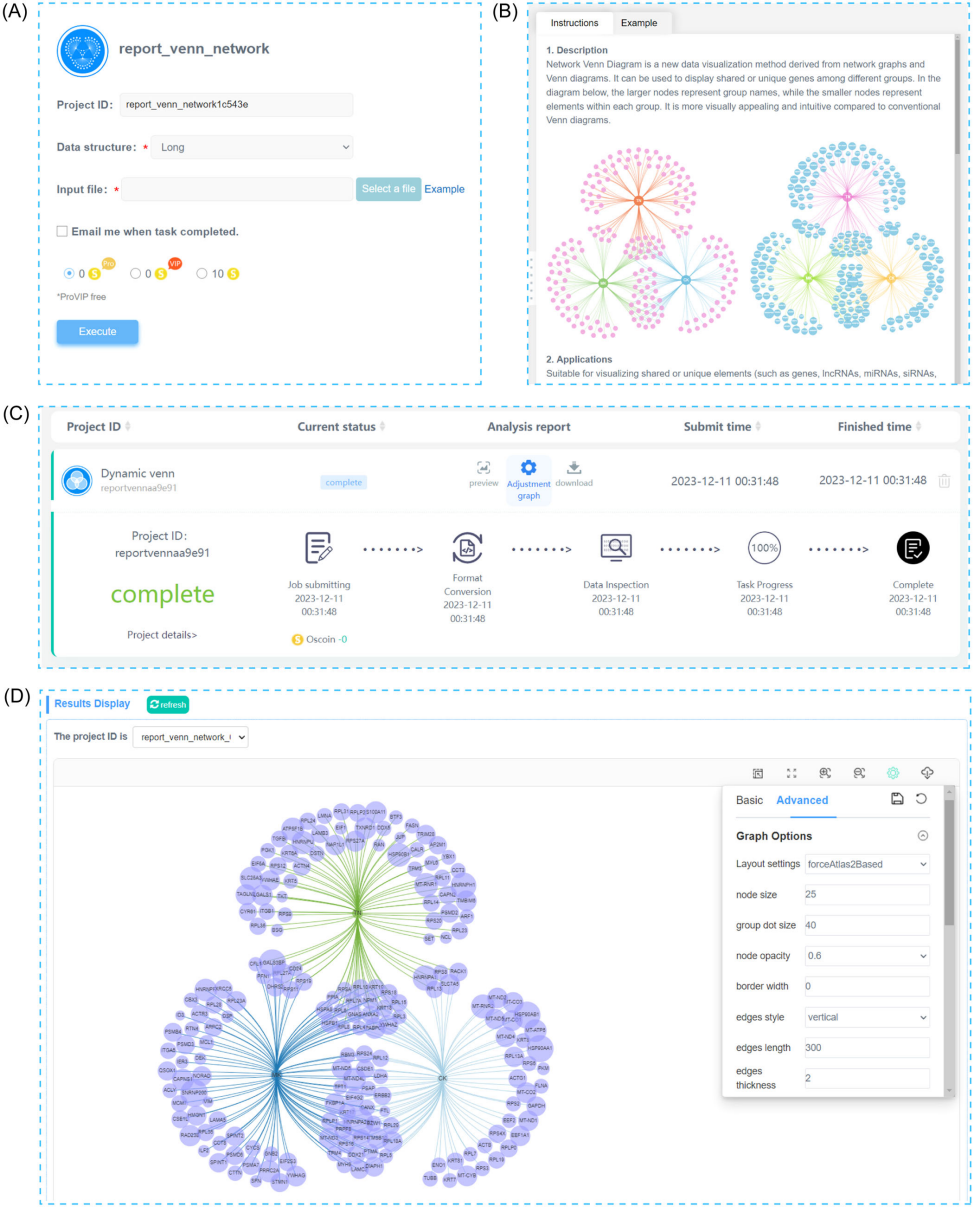
The main functional interface of OmicShare tools. (A) Data submission section; (B) description documentation and sample sections; (C) tasks progress section; (D) plots and graphic toolbar.

The use of OmicShare tools is designed to simplify and enhance the efficiency of analysis. Users begin by selecting the tool that best suits their research needs, uploading their data via a user‐friendly graphical interface, and configuring the necessary parameters to launch their analysis (Figure 2A). The key to a successful analysis is to arrange the data files in a specific format that fulfills the requirements of the data analysis tool.

Each tool within the OmicShare tools is accompanied by a sample data file that is available for download to the users. Users are encouraged to use these sample data files as templates to arrange their own data according to this format to avoid any mistakes (Figure 2B). Proper guidance is provided for setting of the parameter in the tool's instructional documentation. In the case of the many bioinformatics utilities, users are often advised to retain default settings for optimal results.

The OmicShare tools feature a robust monitoring system that tracks task progress and identifies errors. Once an analysis is initiated, users can monitor its progression in real‐time on the project dashboard (Figure 2C). After completion, results are available for immediate preview or download (Figure 2D). In the case of an error, an integrated error‐checking facility provides the potential cause of the error. For additional support, users can consult the detailed documentation or contact the online customer service team. Furthermore, the tools proactively verify the format of data files upon submission. Failure to meet the format criteria will prevent task initiation, triggering a helpful error prompt. OmicShare tools support a wide array of file formats, ensuring broad compatibility with common bioinformatics data types, including FASTA sequence files, tab‐separated or comma‐separated value text files, and Excel spreadsheets.

### Interactive graphical engine for high‐quality pictures

The OmicShare tools possess an advanced interactive graphical engine, crafted with JavaScript to meet the dynamic chart customization needs of the research community. This engine presents users with a range of graphical settings that can be modified in real‐time via an online interface, allowing for tweaks to chart elements including axes, fonts, themes, and color schemes. In addition, the tools have an extensive selection of predefined color schemes, spanning 23 gradient colors and 15 categorized palettes, with the added option for users to concoct their own colors if the defaults are unsatisfactory. For instance, in the dynamic network Venn diagram tool, the graphical adjustments are readily accessible, enabling users to alter the visual aspects of the network nodes and edges, such as shape, size, color, transparency, and label style, or the line color, thickness, curvature, and style (Figure 2D).

These graphical settings are savable, facilitating a swift reinstatement of previous configurations upon revisiting the tool interface or transitioning between tasks. By default, the chart styles in the OmicShare tools are calibrated for publication standards, allowing researchers to produce presentation‐ready results with minimal effort.

Visual outputs from OmicShare tools are primed for inclusion in academic manuscripts, conforming to the visual quality standards of scientific publications. Users are afforded the flexibility to personalize these elements, refining the preset styles to suit their preferences. OmicShare supports the export of both bitmap images (JPG, PNG, BMP, TIF) and vector graphics (PDF, SVG), with the latter allowing for further modifications using graphic editing software such as Adobe Illustrator, Inkscape, or CorelDRAW.

## DISCUSSION

The OmicShare tools suite serves as a zero‐coding, interactive, and user‐friendly platform for online data analysis and visualization, providing a variety of export formats that meet publication standards. These tools facilitate the seamless integration of outputs into scholarly articles by mitigating the steep learning curve commonly associated with complex software. OmicShare tools are renowned for generating heatmaps [11, 12], performing GO and KEGG pathway enrichment analyses [13, 14, 15], conducting PCA [16, 17, 18], and creating Venn diagrams [19, 20]. Since their launch in April 2016, these tools have garnered citations in over 4000 SCI‐indexed papers.

The evolution of OmicShare tools is characterized by continuous enhancements and feature expansions. Despite the robust interactive graphical engine of the OmicShare tools, certain tools exhibit incomplete graphical parameters and lack detailed categorization, necessitating further optimization and adjustment. Future updates are expected to incorporate diverse data types and analytical techniques, such as single‐cell sequencing and clinical data analysis. Concurrently, efforts will be directed toward refining the user interface and parameter settings, thereby enhancing the accessibility of advanced data analysis for researchers, including those with limited bioinformatics expertise.

## METHODS

OmicShare tools, as an online platform, use JavaScript, HTML, and Bootstrap for front‐end development, ensuring a responsive and accessible user interface. On the backend, the advanced web framework ThinkPHP is employed for data preprocessing and statistical analysis. Some tools within OmicShare tools utilize R and Python programming languages for analysis and graphing, incorporating packages such as ggplot2, complexHeatmap, edgeR, and DESeq2; these are integral for conducting complex statistical evaluations and generating high‐quality visualizations. Meanwhile, other tools leverage custom‐developed front‐end drawing plugins, allowing for unique and tailored graphical representations directly based on user data, enhancing the flexibility and user‐specific customization of the platform.

## AUTHOR CONTRIBUTIONS

Huangkai Zhou, Chuan Gao, and Peng Ai conceived the platform and idea. Wenjie Huang and Hongyan Mu designed the software functional modules and graphical user interfaces. Jianzhou Chen, Gui Huang, and Shimiao Hong completed the bioinformatics analysis process, graphical interface, and cloud server development. Hongyan Mu wrote the manuscript. Meiying Deng and Huangkai Zhou were responsible for editing and revising the manuscript. All the authors have read the final manuscript and approved it for publication.

## CONFLICT OF INTEREST STATEMENT

The authors declare no conflict of interest.

## ETHICS STATEMENT

No animals or humans were involved in this study.

## Data Availability

All data are available at https://www.omicshare.com/tools/. Supporting information (scripts, graphical abstract, slides, videos, Chinese translated version, and update materials) may be found in the online DOI or iMeta Science https://www.imeta.science/.
